# Molecular detection of bacterial contamination in plasma using magnetic-based enrichment

**DOI:** 10.1038/s41598-022-12960-5

**Published:** 2022-06-01

**Authors:** Jinyeop Lee, Abdurhaman Teyib Abafogi, Sujin Oh, Ho Eun Chang, Wu Tepeng, Daekyu Lee, Sungsu Park, Kyoung Un Park, Yun Ji Hong

**Affiliations:** 1 School of Mechanical Engineering, Sungkyunkwan University, Suwon, South Korea; 2 KingoBio Inc. Research Center, Suwon, South Korea; 3 Department of Laboratory Medicine, Seoul National University College of Medicine, Seoul, South Korea; 4 PHiCS Institute, Seoul, South Korea; 5 Department of Biophysics, Institute of Quantum Biophysics (IQB), Sungkyunkwan University, Suwon, South Korea; 6 Department of Laboratory Medicine, Seoul National University Bundang Hospital, Seongnam, South Korea

**Keywords:** Biochemistry, Microbiology, Molecular biology, Diseases, Molecular medicine

## Abstract

Bacterial contamination of blood products is a major problem in transfusion medicine, in terms of both morbidity and mortality. Platelets (PLTs) are stored at room temperature (under constant agitation) for more than 5 days, and bacteria can thus grow significantly from a low level to high titers. However, conventional methods like blood culture and lateral flow assay have disadvantages such as long detection time, low sensitivity, and the need for a large volume of blood components. We used real-time polymerase chain reaction (PCR) assays with antibiotic-conjugated magnetic nanobeads (MNBs) to detect enriched Gram-positive and -negative bacteria. The MNBs were coated with polyethylene glycol (PEG) to prevent aggregation by blood components. Over 80% of all bacteria were captured by the MNBs, and the levels of detection were 10^1^ colony forming unit [CFU]/mL and 10^2^ CFU/mL for Gram-positive and -negative bacteria, respectively. The detection time is < 3 h using only small volumes of blood components. Thus, compared to conventional methods, real-time PCR using MNBs allows for rapid detection with high sensitivity using only a small volume of blood components.

## Introduction

Bacterial contamination of blood products is a major problem in transfusion medicine^1–3^. Especially, transfusion of contaminated platelets (PLT) may cause serious infections and septic reactions^1,4^. PLTs are stored at room temperature (under constant agitation) for 5 days; bacteria can thus easily multiply from low levels (< 1 colony forming unit [CFU]/mL) to high titers (< 10^8^ CFU/mL)^5–7^. The US Food and Drug Administration (FDA) reported that transfusion of contaminated PLTs caused 51 deaths from 2001 to 2016^8^. The European Commission reported that 43 transfusions were contaminated in the European Union from 2010 to 2013; 36 involved contaminated PLTs^9^. Although there have been a few cases, the morbidity and mortality rates are very high^10,11^. To reduce mortality, accurate and rapid bacterial detection is required^7^. The gold standard for the detection is blood culture, which is one of the oldest clinical techniques^12,13^. However, bacterial growth to detectable levels usually requires from 24 h^14^ to several days^15^. Also, low bacterial titers and slow bacterial growth can cause false-negative results when automated blood culture systems are employed^16,17^. Much effort has thus been devoted to the rapid and sensitive detection of bacterial pathogens in blood^18–21^. The enhanced bacterial detection (eBDS) system (Haemonetics Corporation, Braintree, MA, USA) indirectly detects bacteria by measuring decreases in oxygen concentration over 24 h, but cannot detect anaerobic bacteria^22^. The Platelet Pan-Genera Detection (PGD) test (Verax Biomedical, Marlborough, MA, USA) is a lateral flow immunoassay detecting lipoteichoic acid (LTA) and lipopolysaccharide (LPS) in aerobic and anaerobic Gram-positive and -negative bacterial species, respectively, within 30 min^23^. However, the sensitivity is low (approximately 10^4^ CFU/mL) and the false-positive rate is high^24^.

Nucleic acid (NA) amplification via polymerase chain reaction (PCR) sensitively and specifically detects bacterial pathogens^25–29^. However, on the day of PLT production, the PCR sensitivity was only 12.8% that of the BacT/ALERT system (Organon Teknika Corp., Durham, NC, USA) because the bacterial loads were very low^30^. There are commercially available molecular diagnostic systems such as T2Bacteria Panel (T2 Biosystems, Lexington, MA, US) and Micro-DxTM (Molzyme, Bremen, Germany) for the identification of patients with symptoms of sepsis. However, no molecular systems have been developed to validate the safety of blood components for transfusion services and conventional products such as PGD test and eBDS system are not related to molecular diagnosis. Also, PLTs contain many substances (such as immunoglobulin G) that interfere with NA amplification^31^. It is thus essential to enrich bacteria and prepare purified bacterial DNA for accurate and rapid detection. Many commercial kits are used to extract NA from blood products; most employ solid-phase extraction^32^. However, these cannot remove inhibitors or enrich pathogens and bacteria are not isolated. Immunomagnetic separation (IMS) using antibody-conjugated magnetic nanobeads (Ab-MNBs) is widely applied to isolate pathogens and thus eliminate inhibitory substances^33–36^. However, Ab-MNBs do not detect all bacteria that cause sepsis; the antibody is specific for 1 species but at least 10 species of bacteria cause sepsis. Thus, MNBs must be conjugated with materials that bind a broad spectrum of bacterial species. Although there have been previous studies using vancomycin conjugated nanobeads to capture several bacterial species, those nanobeads were used in urine and orange juice without molecular detection methods^37–41^.

**Figure 1:**
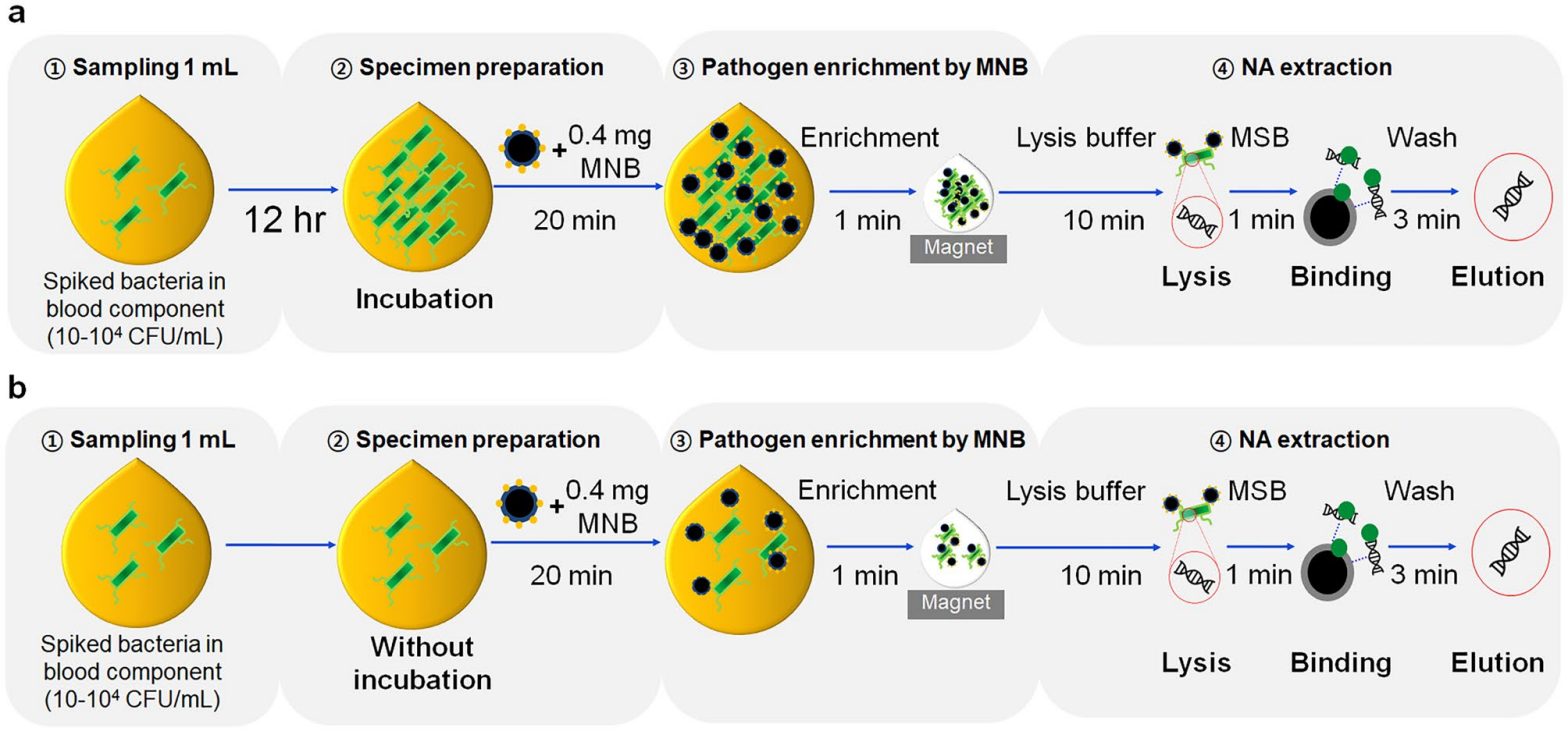
Schematic illustration of pathogen enrichment by MNBs (magnetic nanobeads) and NA (nucleic acid) extraction from 1-mL samples of blood components spiked with bacteria (**a**) after 12 h of specimen incubation at RT (room temperature), and (**b**) without prior specimen incubation. The procedural steps were as follows: sampling, specimen preparation, pathogen enrichment by MNBs, and NA extraction.

Thus, we developed a method that enriches both Gram-positive and -negative bacteria using MNBs coated with two different materials; we then extracted bacterial DNA. Figure 1 shows two methods used for sample preparation (with and without specimen incubation). Performance was tested by spiking 2.5-mL amounts of apheresis plasma with *Escherichia coli* O157:H7 (*E. coli* O157:H7) and *Staphylococcus aureus* (*S. aureus*). Extracted bacterial DNAs were amplified via real-time PCR.

## Results

### Characterization of antibiotics conjugated MNBs

**Figure 2:**
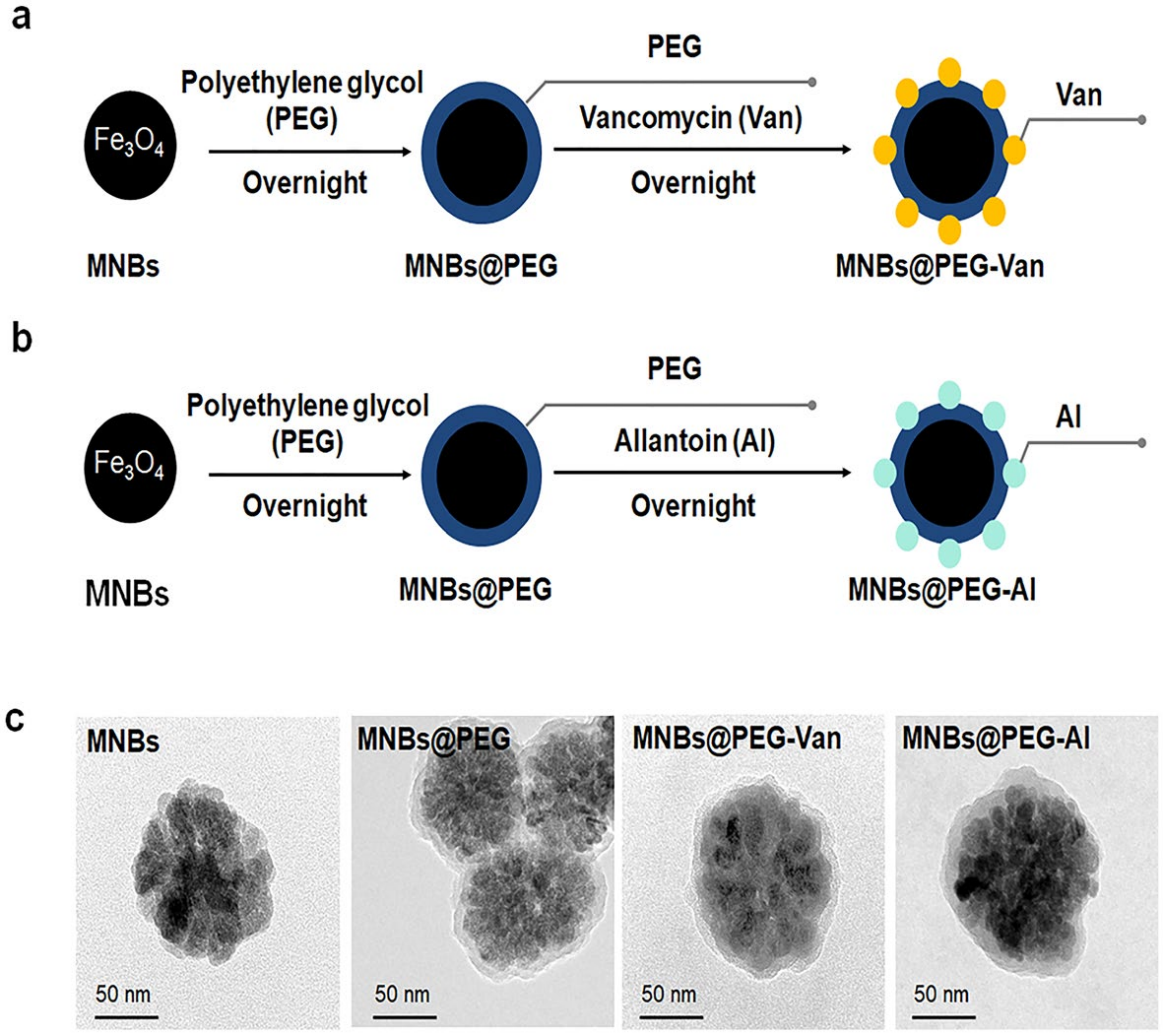
Conjugation of materials to MNBs (magnetic nanobeads). (**a**) Schematic of the synthesis of MNBs@PEG-Van (polyethylene glycol-vancomycin). (**b**) Schematic of the synthesis of MNBs@PEG-Al (polyethylene glycol-allantoin). (**c**) TEM (Transmission electron microscopy) images of unconjugated MNBs, MNBs@PEG, MNBs@PEG-Van, and MNBs@PEG-Al.

The synthesis of antibiotic-conjugated MNBs with PEG coatings is shown in Fig. 2a and b. First, superparamagnetic Fe_3_O_4_ nanobeads with an average diameter of 150 nm were synthesized using a one-step hydrothermal method^42^, coated with PEG, and conjugated with vancomycin (Van) or allantoin (Al) to yield MNBs@PEG-Van and MNBs@PEG-Al. Transmission electron microscopy (TEM) (JSM-3010, JEOL Ltd. Tokyo, Japan) (Fig. 2c) showed that the MNBs had a typical core–shell structure, i.e., a 150-nm-diameter Fe_3_O_4_ core and 5.5-nm-thick PEG coating. The zeta potentials were derived using dynamic light scattering (DLS) (Nano ZS, Malvern Instruments, Malvern, UK). The zeta potential of unconjugated MNBs was −20.33 ± 0.67 mV, whereas those of MNBs@PEG, MNBs@PEG-Al, and MNBs@PEG-Van were −10.58 ± 0.86, −12.40 ± 0.42, and −6.93 ± 0.71, respectively (Supplementary Table S1).

### Efficiencies of bacterial enrichment by MNBs

**Figure 3:**
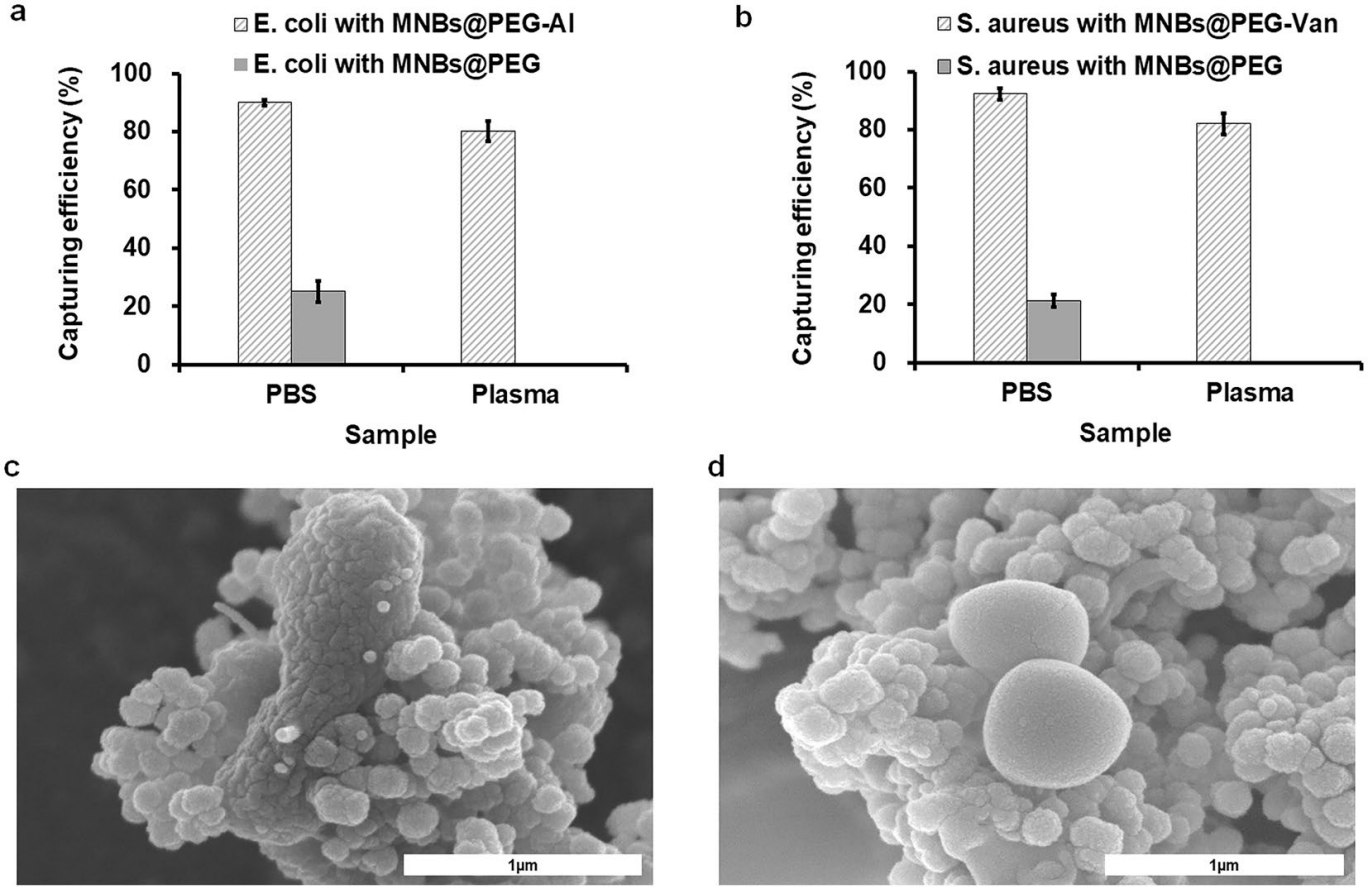
(**a**) Capture efficiencies of *E. coli* with MNBs@PEG-Al (polyethylene glycol-allantoin) and MNBs@PEG from PBS (phosphate-buffered saline) and apheresis plasma. (**b**) Capture efficiencies of *S. aureus* with MNBs@PEG-Van (magnetic nanobeads@polyethylene glycol-vancomycin) and MNBs@PEG from PBS and apheresis plasma. (**c**) FE-SEM (Field-effect scanning electron microscopy) images showing *E. coli* bound to MNBs@PEG-Al. (**d**) FE-SEM images showing *S. aureus* bound to MNBs@PEG-Van.

As shown in Fig. 3a, the MNBs@PEG-Al’s capture efficiencies of *E. coli* spiked in phosphate-buffered saline (PBS) and apheresis plasma were 90% and 80.1%, respectively. The MNBs@PEG-Van’s capture efficiencies of *S. aureus* spiked in PBS and apheresis plasma were 92.3% and 82.1%, respectively (Fig. 3b). Field-effect scanning electron microscopy (FE-SEM) (JSM7500F, JEOL Ltd. Tokyo, Japan) (Fig. 3c and d) showed *E. coli* and *S. aureus* bound specifically to MNBs@PEG-Al and MNBs@PEG-Van, respectively.

### Real-time PCR for the detection of enriched bacteria

**Figure 4:**
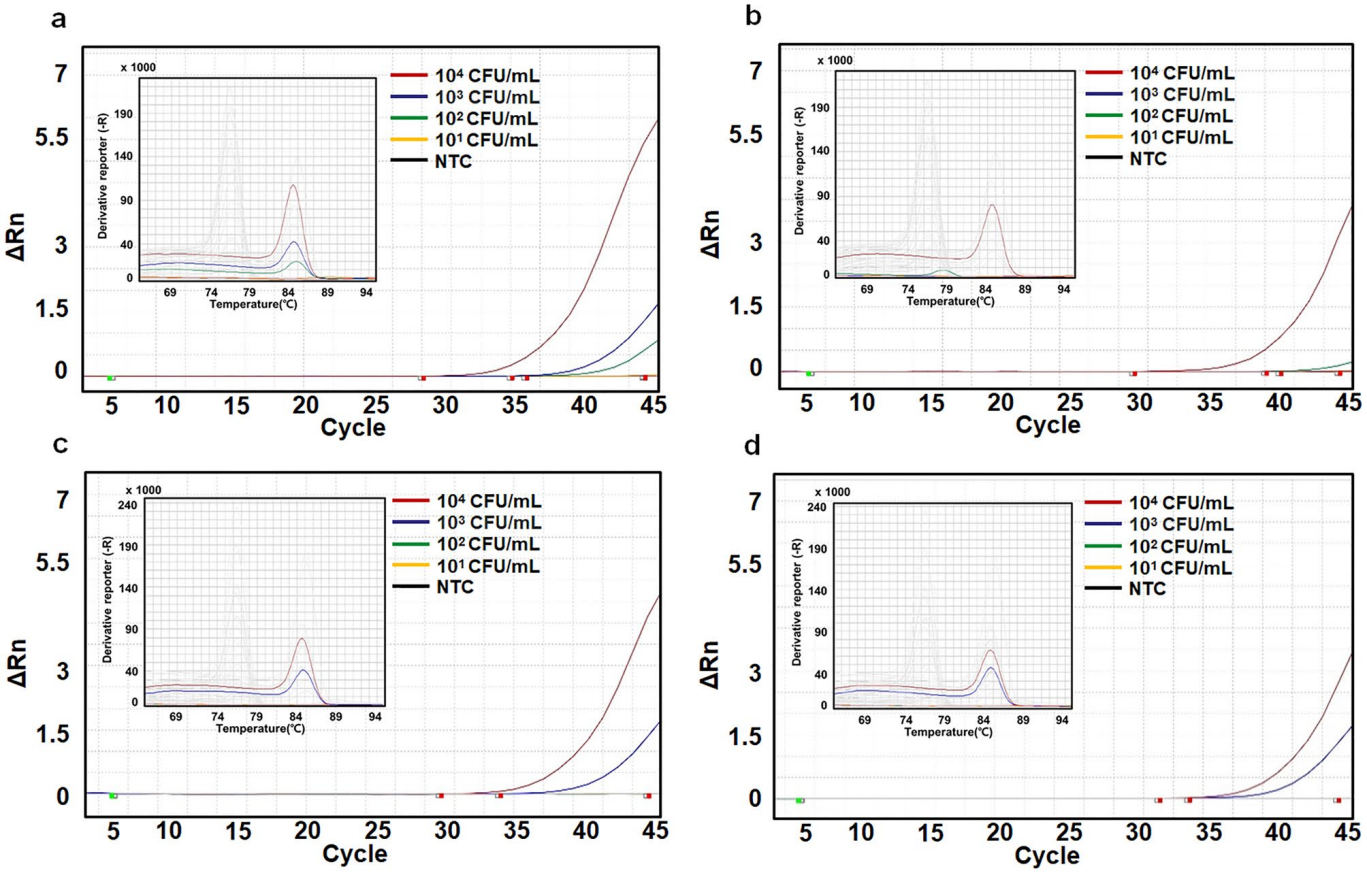
Results of real-time PCR for the detection of *E. coli*. Each figure shows amplification and melting curves. The CFU (colony forming unit) values in each figure are the concentrations at the time of spiking and are independent of specimen incubation. The quantified actual numbers of *E. coli* in plasma with different concentration (10^1^–10^4^ CFU/mL) after 12 h specimen incubation at RT are 98.3 ± 22.4, 741 ± 98, 6,210 ± 1540, and 53,300 ± 12,700, respectively (Supplementary Table S3). Nucleic acid amplification and melting curves in each figure are dependent on specimen incubation. (**a**) Real-time PCR results after 12 h incubation and with *E. coli* captured by MNBs, (**b**) after 12 h incubation and with *E. coli* uncaptured by MNBs, (**c**) without prior specimen incubation and with *E. coli* captured by MNBs, (**d**) without prior specimen incubation and with *E. coli* uncaptured by MNBs.

**Figure 5:**
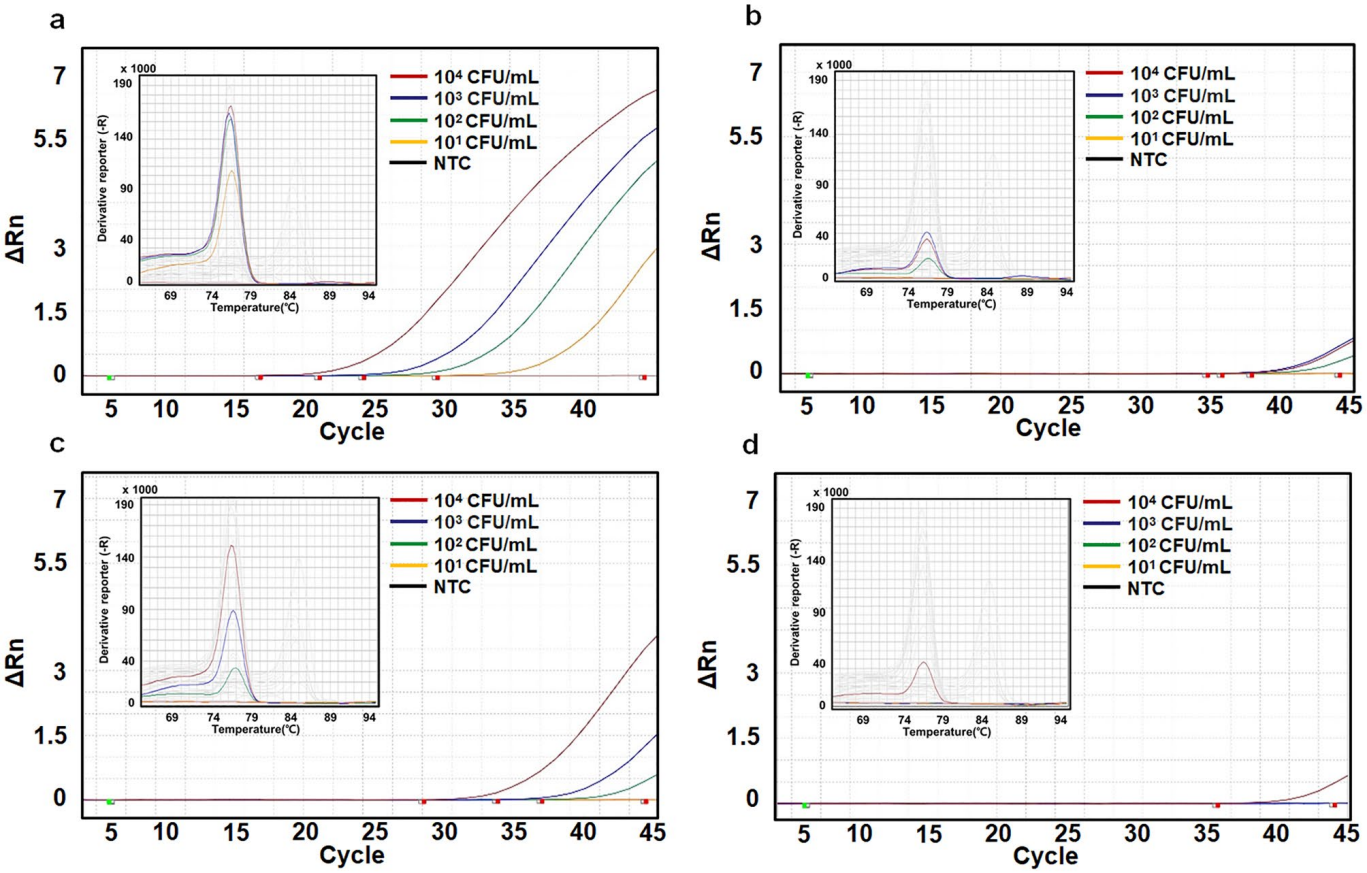
Results of real-time PCR for the detection of *S. aureus*. Each figure shows amplification and melting curves. The CFU (colony forming unit) values in each figure are the concentrations at the time of spiking and are independent of specimen incubation. The quantified actual numbers of *S. aureus* in plasma with different concentration (10^1^–10^4^ CFU/mL) after 12 h specimen incubation at RT are 88.6 ± 14.7, 606 ± 144, 7,210 ± 1070, and 54,000 ± 10,600, respectively (Supplementary Table S3). Nucleic acid amplification and melting curves in each figure are dependent on specimen incubation. (**a**) Real-time PCR results after 12 h incubation and with *S. aureus* captured by MNBs, (**b**) after 12 h incubation and with *S. aureus* uncaptured by MNBs, (**c**) without prior specimen incubation and with *S. aureus* captured by MNBs, (**d**) without prior specimen incubation and with *S. aureus* uncaptured by MNBs.

DNA was extracted from the enriched Gram-negative and -positive bacteria and subjected to real-time PCR. As shown in Fig. 4a, *E. coli* at 10^2^ CFU/mL was detected after 12 h of specimen incubation, and Fig. 4b shows that *E. coli* not captured by MNBs were detected at only 10^4^ CFU/mL. As shown in Fig. 4c, 10^3^ CFU/mL of *E. coli* was detected without prior specimen incubation, but DNA extracted from 10^1^ to 10^2^ CFU/mL could not be amplified. Figure 4d shows that the difference in cycle threshold (Ct) value was approximately 1.4 when apheresis plasma containing *E. coli* captured (Ct; 30.54 ± 0.48) and not captured (Ct; 31.95 ± 0.95) by MNBs at a level of 10^4^ CFU/mL were compared, while MNBs@PEG-Al captured the bacteria effectively at a level of 10^3^ CFU/mL (Table 1). As shown in Fig. 5a, *S. aureus* at 10^1^ CFU/mL was detected after 12 h of specimen incubation. Figure 5b shows that *S. aureus* not captured by MNBs was detected at only 10^4^ CFU/mL. 10^2^ CFU/mL of *S. aureus* was detected without prior specimen incubation, but DNA extracted from 10^1^ CFU/mL could not be detected (Fig. 5c). Figure 5d shows that MNBs@PEG-Van captured the bacteria effectively at a level of 10^4^ CFU/mL.

**Table 1:** Results of real-time PCR analyses.

Bacteria	Incubation	Bacteria captured by MNBs (CFU/mL)								Uncaptured bacteria (CFU/mL)							
		10^4^		10^3^		10^2^		10		10^4^		10^3^		10^2^		10	
		Mean	SD	Mean	SD	Mean	SD	Mean	SD	Mean	SD	Mean	SD	Mean	SD	Mean	SD
*S. aureus*	With prior specimen incubation	18.54	0.20	23.25	0.31	26.09	0.32	31.02	0.23	35.57	0.96	NA	NA	NA	NA	NA	NA
	Without prior specimen incubation	28.21	0.71	34.52	1.10	NA	NA	NA	NA	NA	NA	NA	NA	NA	NA	NA	NA
*E. coli*	With prior specimen incubation	29.07	0.18	33.56	1.09	35.60	0.51	NA	NA	32.37	0.59	NA	NA	NA	NA	NA	NA
	Without prior specimen incubation	30.54	0.48	34.64	0.96	NA	NA	NA	NA	31.95	0.95	NA	NA	NA	NA	NA	NA

CFU, colony forming unit; SD, Standard deviation; NA, not applicable.

## Discussion

When PLTs are stored for 5 days at RT with agitation, even if the initial suspension contains < 1 CFU/mL, the bacteria can proliferate^1,4^. Current culture methods do not yield contamination data during PLT storage. Culture requires 1–5 days and data are available only after the PLTs have been released. Second, a large amount of blood (> 20 mL) is required for culture of both aerobic and anaerobic bacteria^23^. Molecular diagnostics requires small sample volumes (< 1 mL) and detects pathogens within 3 h. It is essential to reduce culture time by improving bacterial enrichment. Such enrichment by MNBs requires that they be dispersible in blood components, and that receptors such as vancomycin and allantoin bind to the bacterial pathogens. Plasma contains many proteins, clotting factors, and IgG^43^; these readily adsorb to non-PEG-coated MNBs. After a 20-min incubation, MNBs aggregated in plasma, but MNBs@PEG did not (Supplementary Fig. S1). PEG prevented non-specific binding to the surfaces of MNBs. The MNBs (4 × 10^9^ to 4 × 10^12^ beads/mL) were added to 1 mL amounts of apheresis plasma containing bacterial pathogens. Over 80% of all *E. coli* and *S. aureus* were captured at MNB levels of 4 × 10^11^ to 4 × 10^12^/mL (Supplementary Table S2). Many bacterial species contaminate PLTs. IMS is not diagnostically useful; the species of bacteria present remain unknown until the results are confirmed. MNBs must be conjugated with receptors that bind to a broad spectrum of bacterial species. Vancomycin binds to the peptidoglycan layer of Gram-positive bacteria that terminate in -Lys-D-Ala-D-Ala^44–46^, and it is known to bind to vancomycin-resistant bacteria^47^. The LPS structure of the outer membrane of Gram-negative bacteria varies depending on the bacterial strain^48^, but it is known that allantoin can bind to most LPS structures regardless of the strain^49^. The capture efficiencies and SEM images showed that these compounds were immobilized on MNBs@PEGs that bound *E. coli* and *S. aureus*, respectively.

According to the current FDA guidance^50^, standard protocol for bacterial contamination of PLT essentially includes incubation for 12 h. Even if there are low cases of bacterial contamination of PLT, but it is essential for safety validation of blood components for public health. Pathogen enrichment by MNBs prior to NA extraction reduced the levels of possible inhibitors and yielded more NA than commercial kits. The detection time was thus dramatically reduced (< 15 h). Both the *E. coli* and *S. aureus* enrichment rates were > 80% and levels of 10^1^ and 10^2^ CFU/mL, respectively, were detected after 12 h of specimen incubation. The levels of detection without incubation were 10^2^ and 10^3^ CFU/mL, respectively. Thus, our method is at least 100-fold more sensitive than lateral flow assay kits such as the Platelet PGD test (limit of detection = 10^4–5^ CFU/mL) after 12 h of specimen incubation^24^. In addition, our method requires only small sample volumes (< 1 mL) for monitoring bacterial contamination. This also means that there is no need to consider the adverse effects of MNBs on PLT as the remaining PLT which is not used for testing will be transfused. However, we aim to further improve the sensitivity and devise a fully automated high-throughput system. Our current focus is on optimization of sample preparation methods for various bacterial strains and their application to other blood components as well.

## Methods

### Bacterial strains

*Escherichia coli* (ATCC 25,922; American Type Culture Collection, Bethesda, MD, USA) and *S. aureus* (ATCC 25,923) were used. A single colony of either strain was transferred to 5 mL of Luria–Bertani (LB) broth (BD, Franklin Lakes, NJ, USA) and cultured for 18 h at 200 rpm and 37 °C. The cultures were then diluted 100-fold with fresh LB broth and incubated at 200 rpm and 37 °C until the optical density at 600 nm (OD_600_) reached 1. Bacterial viability was measured using the standard colony counting method^51^ and suspensions of 10^8^ CFU/mL in PBS (pH 7.4) were prepared.

### Preparation of antibiotics conjugated MNBs

Figure 2a and b show the synthesis of antibiotic conjugated MNBs. Vancomycin and allantoin were obtained from Sigma-Aldrich (St. Louis, MO, USA). The MNBs (100 nm in diameter) were sonicated for about 40 s to prevent aggregation. MNBs (200 mg) were dispersed in 40 mL of 1 M HCl and stirred at room temperature (RT) for 1 h. The MNBs were separated over 2 min using a magnetic rack (Bioneer Co., Ltd, Daejeon, Korea), and residual HCl was removed by washing in 40 mL of PBS three times followed by dispersal in 10 mL PBS. PEG (25 mg) (Sigma-Aldrich) dissolved in 25 mL of Tris buffer (pH 8.5) was mixed with 50 mg MNBs overnight at RT. To conjugate Van to MNBs@PEG-COOH, 5 mg MNBs@PEG-COOH was added to 500 µL of 2-(N-morpholino) ethane sulfonic acid (MES) buffer (0.1 M, pH 6.0) (Sigma-Aldrich); 4 mg ethyl carbodiimide hydrochloride (EDC) (Sigma-Aldrich), 7 mg N-hydroxy succinimide (NHS) (Sigma-Aldrich), and 10 mg vancomycin dissolved in 1 mL of MES buffer were added and the mixture was stirred at RT for 15 min. Then, the MNBs@PEG-COOH suspension was dispersed in the vancomycin-EDC-NHS suspension via continuous stirring at RT for 2 h. MNBs@PEG-Van were separated using the magnetic rack and washed with 500 µL of PBS. Finally, MNBs@PEG-Van were resuspended in 1 mL of PBS and stored at 4 °C. MNBs@PEG-Al were similarly prepared.

### Characterization of antibiotics conjugated to MNBs

MNBs were dispersed in 99.5% (v/v) ethanol at 20 μg/mL. Copper grids bearing carbon films (Electron Microscopy Sciences, Hatfield, PA, USA) were immersed in 1-mL MNB dispersions for 10 min, removed using tweezers, dried at 70 °C, and examined by TEM (JEOL Ltd.) operating at 200 kV.

### Bacterial enrichment by MNBs

Apheresis plasma containing bacteria (*E. coli* O157:H7 and *S. aureus*) at 10^1^–10^4^ CFU/mL were incubated at RT for 12 h. One milliliter of plasma was mixed with 200 μL of either MNBs@PEG-Al or MNBs@PEG-Van (4 × 10^11^ particles/mL, final concentration) and the mixtures were incubated at RT for 20 min. Bacteria-MNB clusters were separated using a magnetic rack. The residues, which contained uncaptured bacteria, were transferred to 1.5-mL tubes. The MNBs were washed with 1 mL PBS twice and dispersed in 200 µL PBS.

### FE-SEM of bacteria binding to antibiotics conjugated MNBs

Bacteria-enriched MNBs were washed twice with PBS and MNBs separated on a magnetic rack. Fixation [in 2% (w/v) glutaraldehyde] proceeded at RT for 1 h. MNB-bacteria complexes were washed three times with 1 mL PBS, incubated with 1% (w/v) osmium tetroxide for 1 h at 4 °C in the dark, and washed three times with PBS followed by gradual dehydration in ethanol (30, 50, 70, 80, 90, and 99.5% [v/v]) for 10 min each time. Ten microliters of MNB-bacteria complexes were dropped onto copper grids covered with amorphous carbon and dried at RT for 2 h. FE-SEM (JEOL Ltd.) was used to image the complexes.

### NA extraction

MagListo 5 M Genomic NA extraction kits (Bioneer) were used to extract bacterial DNA as suggested by the manufacturer. Two hundred microliters of lysis buffer were added to a suspension of BE-MNBs followed by incubation for 10 min at RT. The elution volume was 100 µL; the extracted DNA was stored at −80 °C. DNA purity and yield were assessed by quantifying absorbance at 230, 260, and 280 nm using a Nano-200 spectrophotometer (Allsheng, Hangzhou, China). To detect Gram-positive bacteria, 10 µg lysostaphin (Sigma-Aldrich) was added to the BE-MNBs followed by incubation for 10 min at 37 °C prior to the addition of lysis buffer.

### Real-time PCR assay

Real-time PCR was used to confirm the identities of the captured bacteria. The primers were designed using PrimerQuest (Integrated DNA Technologies Inc., Coralville, IA, USA): SA nuc_F (5′-TATGGACGTGGCTTAGCGTAT-3′) and SA nuc_R (5′-GACCTGAATCAGCGTTGTCTT-3′) for *S. aureus*; and EB tyrB_F (5′-AAGAGGATGCCTACGCCATT-3′) and EB tyrB_R (5′-CTTGGCGGGCTGGAGTAGTT-3′) for *E. coli*. Power SYBR Green PCR Master Mix (Applied Biosystems, Waltham, MA, USA) served as the PCR master mix; all primers were added to 0.2 µM. QuantStudio 3 (Applied Biosystems) was used to perform PCR. Positive and negative amplification controls were included in every run. The positive controls contained DNA directly extracted from *S. aureus* and *E. coli*, and the negative control was RNase- and DNase-free water. A result was considered positive when the threshold cycle (Ct) was > 37.0 and the melting temperature (Tm) was appropriate (76.0 ± 0.5 °C).

## Supplementary Information

Supplementary Information.

## Data Availability

The data supporting this study are included in this published article and its Supplementary Information.
