# Supplementary material for: Molecular detection of bacterial contamination in plasma using magnetic-based enrichment
Source: Sci Rep. 2022 Jun 1;12:9151. doi: 10.1038/s41598-022-12960-5 (PMC9160056; doi:10.1038/s41598-022-12960-5)

**Supplementary Information for**

Molecular detection of bacterial contamination in Plasma using magnetic-based enrichment

Jinyeop Lee^1,2†^, Abdurhaman Teyib Abafogi^1†^, Sujin Oh^3^, Ho Eun Chang^4^, Wu Tepeng^1,2^, Daekyu Lee^1^, Sungsu Park^1,5*^, Kyoung Un Park^3,6^, Yun Ji Hong^3,6*^

* SP (nanopark@skku.edu, +82-31-290-7431); YJH (aeiea@snu.ac.kr, +82-31-787-7695)

**This Word file includes:**

Figure S1

Table S1 to S3

**Table S1.** Zeta potential measurement of four different magnetic bead types including magnetic nanobeads (MNBs), MNBs@polyethylene glycol (PEG), MNBs@PEG-Allantoin (Al), and MNBs@PEG-Vancomycin (Van).

| Bead types | Zeta potential (mV) |
| --- | --- |
| MNBs | -20.33 ± 0.67 |
| MNBs@PEG | -10.58 ± 0.86 |
| MNBs@PEG-Al | -12.40 ± 0.42 |
| MNBs@PEG-Van | -6.93 ± 0.71 |

**Table S2.** The capturing efficiency of two different species of bacteria in plasma with different concentration (4 ⅹ10^9^ - 4 ⅹ10^12^) of the magnetic beads (MNBs) for capturing the bacteria. There was no statistically significant difference in capturing efficiency between the 4 ⅹ10^11^ - 4 ⅹ10^12^ beads/mL and these two groups were tested by student’s t-test.

| Species (n=3) | Bacteria capturing efficiency (%) | | | |
| --- | --- | --- | --- | --- |
|  | 4 ⅹ10^9^ beads/mL | 4 ⅹ10^10^ beads/mL | 4 ⅹ10^11^ beads/mL | 4 ⅹ10^12^ beads/mL |
| *E. coli* | 50.3 ± 4.4 | 60.1 ± 3.4 | 80.1 ± 3.4 | 83.3 ± 2.4 |
| *S. aureus* | 40.6 ± 4.7 | 50.6 ± 4.4 | 82.1 ± 3.7 | 84 ± 3.4 |

**Table S3.** The quantified actual numbers of two different species of bacteria in plasma with different concentration (10^1^ – 10^4^ CFU/mL) without incubation and after 12 h specimen incubation at RT.

|  | **Species (n=3)** | **Bacteria concentration (CFU/mL)** | | | |
| --- | --- | --- | --- | --- | --- |
|  |  | x 10^1^ | x 10^2^ | x 10^3^ | x 10^4^ |
| Timepoint of spiking  (without incubation) | *E. coli* | 2.3 ± 0.5 | 2.8 ± 0.3 | 2.2 ± 0.4 | 2.4 ± 0.5 |
|  | *S. aureus* | 2.6 ± 0.6 | 2.2 ± 0.3 | 2.1 ± 0.7 | 2.5 ± 0.6 |
| Timepoint of testing  (after 12 h incubation) | *E. coli* | 98.3 ± 22.4 | 74.1 ± 9.8 | 62.1 ± 15.4 | 53.3 ± 12.7 |
|  | *S. aureus* | 88.6 ± 14.7 | 60.6 ± 14.4 | 72.1 ± 10.7 | 54 ± 10.6 |

**Figure S1.** Effect of PEG coating on preventing aggregation of MNBs (magnetic nanobeads) in apheresis plasma. Both MNBs and MNBs@PEG (polyethylene glycol) were mixed with 1 mL of apheresis plasma at RT for 20-min incubation respectively and these beads were washed with 1 mL of PBS 1 times.


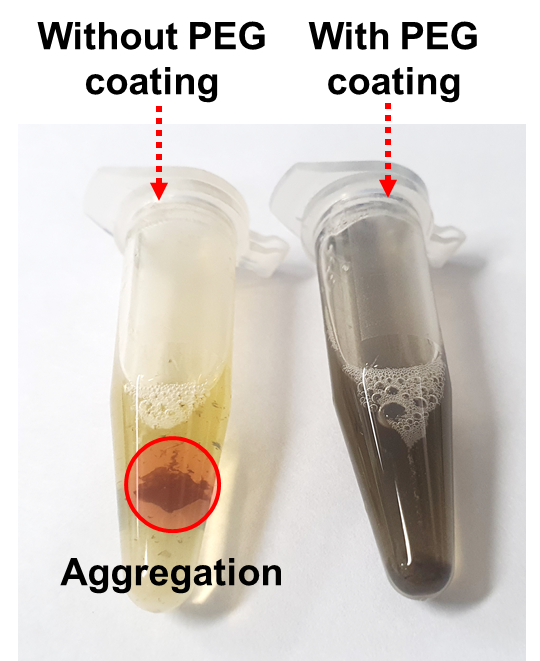

Supplement: Supplementary file 1 — Supplementary Information. [file 41598_2022_12960_MOESM1_ESM.docx]
